# The Effects of the Combination of Mesenchymal Stromal Cells and Nanofiber-Hydrogel Composite on Repair of the Contused Spinal Cord

**DOI:** 10.3390/cells11071137

**Published:** 2022-03-28

**Authors:** Agnes E. Haggerty, Ines Maldonado-Lasunción, Yohshiro Nitobe, Kentaro Yamane, Megan M. Marlow, Hua You, Chi Zhang, Brian Cho, Xiaowei Li, Sashank Reddy, Hai-Quan Mao, Martin Oudega

**Affiliations:** 1 The Miami Project to Cure Paralysis, University of Miami, Miami, FL 33136, USA; ahaggerty@akoyabio.com (A.E.H.); imaldola@gmail.com (I.M.-L.); n1t0bey0sh1r0@gmail.com (Y.N.); woodblocks0311@gmail.com (K.Y.); mmarlow135@yahoo.com (M.M.M.); 2 Department of Regeneration of Sensorimotor Systems, Netherlands Institute for Neuroscience, Institute of the Royal Netherlands Academy of Arts and Sciences, 1105 BA Amsterdam, The Netherlands; 3 Shirley Ryan AbilityLab, Chicago, IL 60611, USA; 4 Department of Physical Therapy and Human Movements Sciences, Northwestern University, Chicago, IL 60611, USA; 5 Department of Orthopedic Surgery, Hirosaki University Graduate School of Medicine, Hirosaki 036-8562, Japan; 6 Department of Orthopedic Surgery, Okayama University Graduate School of Medicine, Dentistry, and Pharmaceutical Science, Kitaku, Okayama 700-8558, Japan; 7 Department of Oncology and Hematology, Affiliated Cancer Hospital & Institute of Guangzhou Medical University, Guangzhou 510095, China; youhua307@163.com; 8 Translational Tissue Engineering Center, Johns Hopkins School of Medicine, Baltimore, MD 21231, USA; czhang96@jhu.edu (C.Z.); bcho8@uw.edu (B.C.); xiaoweili@wustl.edu (X.L.); 9 Department of Materials Science and Engineering, Johns Hopkins University, Baltimore, MD 21218, USA; 10 Institute for NanoBioTechnology, Johns Hopkins University, Baltimore, MD 21218, USA; sreddy6@jhmi.edu; 11 Department of Plastic and Reconstructive Surgery, Johns Hopkins School of Medicine, Baltimore, MD 21287, USA; 12 Division of Plastic and Reconstructive Surgery, Department of Surgery, Washington University School of Medicine, St. Louis, MO 63110, USA; 13 Department of Biomedical Engineering, Johns Hopkins University, Baltimore, MD 21205, USA; 14 Department of Neuroscience, Northwestern University, Chicago, IL 60611, USA; 15 Edward Hines Jr. VA Hospital, Hines, IL 60141, USA

**Keywords:** nanofiber-hydrogel composite, spinal cord injury, inflammation, macrophages, secondary injury, astrocytes, axon growth

## Abstract

A bone marrow-derived mesenchymal stromal cell (MSC) transplant and a bioengineered nanofiber-hydrogel composite (NHC) have been shown to stimulate nervous tissue repair in the contused spinal cord in rodent models. Here, these two modalities were combined to assess their repair effects in the contused spinal cord in adult rats. Cohorts of contused rats were treated with MSC in NHC (MSC-NHC), MSC in phosphate-buffered saline (MSC-PBS), NHC, or PBS injected into the contusion site at 3 days post-injury. One week after injury, there were significantly fewer CD68+ cells in the contusion with MSC-NHC and NHC, but not MSC-PBS. The reduction in CD86+ cells in the injury site with MSC-NHC was mainly attributed to NHC. One and eight weeks after injury, we found a greater CD206+/CD86+ cell ratio with MSC-NHC or NHC, but not MSC-PBS, indicating a shift from a pro-inflammatory towards an anti-inflammatory milieu in the injury site. Eight weeks after injury, the injury size was significantly reduced with MSC-NHC, NHC, and MSC-PBS. At this time, astrocyte, and axon presence in the injury site was greater with MSC-NHC compared with MSC-PBS. We did not find a significant effect of NHC on MSC transplant survival, and hind limb function was similar across all groups. However, we did find fewer macrophages at 1 week post-injury, more macrophages polarized towards a pro-regenerative phenotype at 1 and 8 weeks after injury, and reduced injury volume, more astrocytes, and more axons at 8 weeks after injury in rats with MSC-NHC and NHC alone compared with MSC-PBS; these findings were especially significant between rats with MSC-NHC and MSC-PBS. The data support further study in the use of an NHC-MSC combination transplant in the contused spinal cord.

## 1. Introduction

The prevalent mechanism of spinal cord injury (SCI) in humans is a contusion, which typically leads to nervous tissue damage and sensory and motor function loss [1,2]. The limited endogenous repair of central nervous tissue and typically poor recovery of function after a spinal cord contusion in humans motivates the ongoing search for reparative treatments [1,3,4].

Studies in animal models of contusive SCI have shown that a transplant of bone marrow-derived mesenchymal stromal cells (MSC) in the injury site elicited nervous tissue repair and, albeit not in all cases, function improvements [5,6,7,8,9,10,11]. MSC secrete paracrine factors [12,13,14] that can direct immunomodulatory effects [15,16,17,18], promote neuroprotection [12,13], and increase axon growth [19,20]. Transplanting MSC is considered a promising strategy to treat SCI, but the overall effects of an MSC transplant on nervous tissue repair in the injured spinal cord remain limited [6,9,21].

We demonstrated that an in situ forming, injectable nanofiber-hydrogel composite (NHC) elicited nervous tissue repair in an adult rat model of contusive SCI [22]. The NHC consists of poly(ε-caprolactone) (PCL) nanofibers interfacially bound to a hydrogel network formed from thiolated hyaluronic acid (HA-SH) and poly(ethylene glycol) diacrylate (PEGDA). The composite has a shear storage modulus similar to that of the nervous tissue in the spinal cord [23,24] and provides a sufficiently high porosity to support host cell infiltration and migration [22,25]. Importantly, we showed that NHC modulated the inflammatory response towards anti-inflammatory, pro-regenerative, macrophage phenotypes, and facilitated tissue formation in the injury site [22].

In the present study, we combined two repair modalities by using the NHC as a transplant matrix for MSC. We investigated the combination’s therapeutic potential in the contused adult rat thoracic spinal cord. The repair was assessed by evaluating immunomodulation, neuroprotection, and hindlimb locomotor function.

## 2. Materials and Methods

### 2.1. Animals

Adult female Sprague Dawley rats (*n* = 68, 200–220 g, Charles Rivers Laboratory; Wilmington, MA, USA) were used in this study. All animal procedures were performed according to the guidelines of the National Institutes of Health and the United States Department of Agriculture at the Miami Project to Cure Paralysis at the University of Miami, Miami, FL, and approved by the local Institutional Animal Care and Use Committee. The Assessment and Accreditation of Laboratory Animal Care accredited the animal facility where the procedures were performed. Pairs of rats were housed under a 12-h light/dark cycle with ad libitum access to food and water.

### 2.2. NHC Preparation

NHC was made following the protocol previously described [22]. Briefly, nanofibers were electrospun from a PCL solution (16% *w/w*; Sigma-Aldrich, St. Louis, MO, USA) in a mixture of dichloromethane (Sigma-Aldrich) and dimethylformamide (9/1, *v/v*; Sigma-Aldrich). Poly(9,9-dioctylfluorene-alt-benzothiadiazole) (F8BT; Sigma-Aldrich), a green fluorescent dye, was added to the PCL solution to enable fiber identification after injection [22]. Carboxyl groups were introduced to the surface of the fibers in a plasma cleaner (expanded plasma cleaner; PDC-001; Harrick Plasma; Ithaca, NY, USA). These carboxyl groups were initiated by ethyl dimethylaminopropyl carbodiimide (Sigma-Aldrich) and N-hydroxysuccinimide (Sigma-Aldrich) and then converted to maleimide (MAL) groups by N-(2-aminoethyl) maleimide (Sigma-Aldrich). The MAL-modified fibers were cryogenically milled, sterilized with 70% ethanol. All three components of NHC were stored individually at −20 °C, and thawed 30 min prior to use. NHC was prepared by mixing MAL-modified fibers (10 mg/mL) into a mix of HA-SH (4 mg/mL; ESI BIO, Alameda, CA) and PEGDA (2 mg/mL; ESI BIO) in sterile phosphate-buffered saline (PBS) [22,25] on ice. We mixed and injected NHC or MSC-NHC within 15 min after exposing the contused spinal cord (see below Section 2.4). Once mixed, NHC can be kept on ice for 6 h for injections.

### 2.3. MSC Preparation

MSCs were harvested from bone marrow from femurs and tibias of adult female Sprague Dawley rats (*n* = 6) following a previously described protocol [9,26]. MSCs were cultured on poly-D-lysine-coated dishes in Dulbecco’s Modified Eagle Medium (DMEM; Sigma-Aldrich) with 10% fetal bovine serum (Mediatech; Herndon, VA, USA), 0.03% L-glutamine (Sigma-Aldrich), and 0.1% gentamycin (VWR; Radnor, PA, USA). The cells were transduced with lentiviral vectors encoding for green fluorescent protein (GFP) at a multiplicity of infection of 100, passaged 4 times, and then harvested for transplantation [9]. MSC remained on ice until mixing with freshly prepared NHC or sterile PBS prior to injection into the epicenter of the spinal cord contusion.

### 2.4. Spinal Cord Contusion and Injection

Rats were anesthetized using an intraperitoneal injection of ketamine (50 mg/kg; Zoetis; Parsippany, NJ, USA) and dexmedetomidine (0.5 mg/kg; Zoetis). In the absence of corneal, hindlimb, and tail pinch reflexes, the rats were shaved and cleaned with Nolvasan^®^ skin and wound cleaner (Zoetis). Refresh^®^ Lacri-Lube^®^ eye ointment (Allergan; Madison, NJ, USA) was applied to the eyes to prevent drying during surgery. The 9th thoracic spinal cord segment was exposed and impacted onto its dorsal midline with a force of 175 kDyne using the Infinite Horizon IH-0400 impactor (Precision Systems and Instrumentation LLC; Versailles, KY, USA) [27] resulting in a bilateral injury, as previously described [9,10,28]. After rinsing the injury site with sterile lactated Ringers’ solution containing 0.1% gentamicin (VWR), the muscles were sutured, and the skin closed with Michel wound clips (Fine Science Tools; Foster City, CA, USA). The rats received a subcutaneous injection of the α2-adrenergic receptor antagonist, atipamezole (antisedan; 1.5 mg/kg; Zoetis) to reverse the sedative and analgesic effects of dexmedetomidine. Laboratory personnel monitored the rats until fully awake and applied after-surgery treatments as previously described [9,10].

At 3 days after injury, rats were anesthetized using an intraperitoneal injection of ketamine (50 mg/kg; Zoetis) and dexmedetomidine (0.5 mg/kg; Zoetis). The contused spinal cord was exposed and the injury epicenter was injected with a total volume of 5 µL with 500,000 MSC in NHC or PBS, or with NHC or PBS only at a rate of 1 µL/min using a 10 µL Hamilton syringe, fitted with a pulled glass needle, fixed to a stereotaxic device [6,9]. The internal diameter of the pulled glass needles was, on average, 120 µm; no clogging of the needle was observed during the injections. The needle was kept in place for 5 min after the injection was completed to prevent backflow. Possible leakage was verified using a UV flashlight to detect green fluorescence on the spinal cord. Note that for NHC, the individual components were mixed and injected within 15 min after exposing the contused spinal cord. For MSC-NHC, the NHC was mixed first and then the MSC was mixed into the NHC and injected within 15 min after exposing the contusion site. After the injection was completed, the muscles were sutured, and the skin was closed with Michel wound clips (Fine Science Tools). The rats received an intraperitoneal injection of antisedan (1.5 mg/kg; Zoetis) to reverse the anesthesia. Laboratory personnel monitored the rats until fully awake and applied after-surgery treatments as previously described [9,10]. Each of the four experimental groups contained 15 rats, which survived for 1 week (*n* = 5) or 8 weeks (*n* = 10) after injury. The rats that survived for 1 week were fixed (see histological preparations) and their spinal cord was used for histological and anatomical assessments. The rats that survived for 8 weeks were used to evaluate hind limb motor function during survival (*n* = 10) and, after fixation (see histological preparations), for histological and anatomical assessments (*n* = 5; randomly selected). Two rats died during surgery and were replaced.

### 2.5. Assessment of Hind Limb Function

Hind limb overground walking was assessed weekly using the Basso, Bresnahan, Beattie (BBB) open-field walking test [9,29]. Individual values were averaged per experimental group per time point. In addition, we assessed hind limb sensorimotor function at 4 and 8 weeks after injury using the horizontal ladder test [9,30,31]. For this, personnel blinded to the treatment used high-definition video recordings of the ladder crossings to enable accurate quantification of foot and leg slips, which were qualified as medium or large slips, respectively. The total sum of medium and large slips was expressed as a percentage of the number of steps taken to cross the ladder and averaged per rat and per group for each time point.

### 2.6. Histological Preparations

At 1 and 8 weeks after injury, rats were deeply anesthetized and transcardially perfused with 300 mL of PBS followed by 400 mL of 4% paraformaldehyde in PBS [9]. We dissected the spinal cord from the rats and kept them in the same fixative overnight, followed by 30% sucrose in PBS for 2 days. We then removed a 12 mm long segment centered on the contusion of each spinal cord and embedded these in frozen section medium (NEG 50; Richard-Allan Scientific, Thermo Fisher Scientific, Kalamazoo, MI, USA). The embedded frozen segments were cut into twelve series of 20 µm thick horizontal sections on a cryostat (CM 1950; Leica Biosystems; Buffalo Grove, IL, USA). Each series represented the width of the spinal cord with sections at 240 µm intervals. The sections were collected on glass slides and stored at −20 °C until staining.

### 2.7. Immunohistochemistry

For immunostaining, 20 µm thick tissue sections were rinsed for 5 min in PBS and incubated in PBS with 5% normal goat serum and 0.3% Triton X-100 for 1 h at room temperature to block non-specific antibody binding and permeabilize the tissue. Next, the sections were incubated with primary antibodies (Table 1) mixed in PBS with 5% normal goat serum and 0.3% Triton X-100 for 2 h at room temperature, followed by overnight at 4 °C. Subsequently, the sections were rinsed 3 times for 5 min in PBS and then incubated with goat secondary antibodies (Alexa Fluor 488, Alexa Fluor 555, or Alexa Fluor 647; 1:500; Invitrogen; Carlsbad, CA, USA) against the host of the primary antibody, mixed in PBS, for 2 h at room temperature. After this incubation, the sections were rinsed 3 times for 5 min in PBS, counterstained for 3 min with the nuclear marker, 4′,6-diamidino-2-phenylindole (DAPI; ThermoFisher Scientific; Waltham, MA, USA), rinsed 2 times for 5 min in PBS, and then covered with a glass slip with fluorescence mounting medium (DAKO; Agilent; Santa Clara, CA, USA). The 3 middle sections of the contusion site in a series were identified and imaged using the Olympus VS120 slide scanner with 10× (UPISAPO, 0.4NA, Air) or 20× (UPISAPO, 0.75NA, Air) objectives and fitted with a Hamamatsu ORCA-Flash 4.0 camera (Hamamatsu; Bridgewater, NJ, USA). Autofocus was set on the DAPI channel for cell quantification, and on the axon marker channel for axon quantification.

### 2.8. Injury Volume Assessment

For measuring the injury volume, we used the ImageJ measure function on sections stained for glial fibrillary acidic protein (GFAP) at 1 and 8 weeks after injury. The injury site was defined by the inner border of the surrounding GFAP+ scar. The injury site volume was determined by taking the surface area of the injury site in the middle section, multiplied by the known distance between sections, and adding up the surface area of the edge sections multiplied by 1/2 of the known distance between sections [22]. The individual injury volumes were averaged per experimental groups.

### 2.9. Automated Quantification

An ImageJ Find Maxima plugin (modified from [32]) was used for the automated quantification of cells and axons. Images were corrected for the background by subtracting a Gaussian filtered image, converted to 8-bit, and thresholded to create a count mask. For CD86+ or CD206+ macrophages, two thresholded images from the general macrophage marker, CD68, and from each of the polarization markers were used to create double count masks, each overlaying the thresholded DAPI filter image. For GFP+ MSC, a thresholded image from GFP was used to create a count mask to overlay the thresholded DAPI filter image. Particles that were positive for both the count mask and DAPI were summed to determine the total count of positive hits. The average particle size of ten randomly selected nuclei for the specific cell of interest was used to create a multiplication factor to account for multiple overlaying nuclei. Total counts were multiplied by the multiplication factor to acquire standardized counts, which were averaged per rat and per experimental group.

### 2.10. Macrophage Quantification

We used automated quantification to evaluate CD68+, CD86+, and CD206+ macrophages in the spinal cord at 1 and 8 weeks after injury. Quantification was performed in each of the middle three sections of the contusion, in a 1.5 × 2 mm region of interest centered on the midpoint of the injury. This selected 2 mm long and 1.5 mm wide region of interest covered the injury site for 1 mm caudal and rostral from the midpoint of the contusion. CD68 is a pan-macrophage marker, CD86 is a pro-inflammatory macrophage marker, and CD206 is an anti-inflammatory macrophage marker. While recognizing the range of phenotypes among macrophages within an injury site, we used CD86 to indicate a pro-inflammatory (M1-like) macrophage phenotype and CD206 to indicate an anti-inflammatory (M2-like) macrophage phenotype. We determined cell segmentation counts (DAPl+) residing within double-positive (CD86+/CD68+ and CD206+/CD68+) masks. We standardized the counts by dividing the average particle size by the average size of 10 nuclei of each of the macrophage populations to create a multiplication factor to account for multiple overlaying nuclei. We reported our particle counts multiplied by the multiplication factor as the standardized average count, which were averaged per rat and then per group. We determined the total number of macrophages and the CD206/CD86 ratio. Counts were averaged per rat and per experimental group.

### 2.11. Astrocyte Presence Examination

Automated quantification was used to evaluate GFAP+ astrocytes in the injured spinal cord at 1 and 8 weeks after injury. Quantification was performed in each of the middle three sections of the contusion, in a 2.5 × 4 mm region of interest centered on the midpoint of the injury. This selected 4 mm long and 2.5 mm wide region of interest covered the injury site and adjacent glia scar for 2 mm caudal and rostral from the midpoint of the contusion. The total area and the percent area of GFAP were determined using the batch threshold and measure function of ImageJ. Measurements were averaged per rat and per experimental group.

### 2.12. Axon Presence

We used automated quantification to evaluate axons, recognized with antibodies against neurofilament high molecular weight (NFh), in the contusion at 8 weeks after injury. Quantification was performed in each of the middle three sections of the contusion, in a 1.5 × 2 mm region of interest centered on the midpoint of the injury. This selected 2 mm long and 1.5 mm wide region of interest covered the injury site for 1 mm caudal and rostral from the midpoint of the contusion. The total area and the percent area positive for NFh were determined at 8 weeks after injury. Standardized ImageJ algorithms were used for batch thresholding of all images, and all measurements were averaged per rat and per experimental group.

### 2.13. Transplant Survival Assessment

Automated quantification was used to quantify GFP+ MSC in the contusion at 1 and 8 weeks after injury. Quantification was performed in each of the middle three sections of the contusion, in a 2 × 2 mm region of interest centered on the midpoint of the injury. This selected 2 mm long and 2 mm wide region of interest covered the injury site for 1 mm caudal and rostral from the midpoint of the contusion. Positive particles for the GFP count mask and DAPI were quantified to determine the total number of positive hits. We standardized the counts by dividing the average particle size by the average size of 10 nuclei of GFP+ MSC to create a multiplication factor to account for multiple overlaying nuclei. We reported our particle counts multiplied by the multiplication factor as the standardized average count, which were averaged per rat and per experimental group.

### 2.14. Statistical Analyses

Data were shown as means ± SEM (standard error of the mean). For statistical analyses, we used IBM SPSS Statistics for Windows, version 24 (IBM; Armonk, NY, USA). Data were analyzed by nonparametric ANOVA (independent-samples Kruskal–Wallis test) or parametric (or nonparametric) repeated-measures ANOVA (Friedman’s test), followed by Bonferroni post hoc correction, unless otherwise stated. Pearson’s correlation analysis was used to determine the relationship between variables expressed by the coefficient of determination (r^2^) and considered strong when r^2^ > 0.5. Differences were accepted as statistically significant if *p* < 0.05.

## 3. Results

### 3.1. Inflammatory Response in the Injury Site

**Figure 1:**
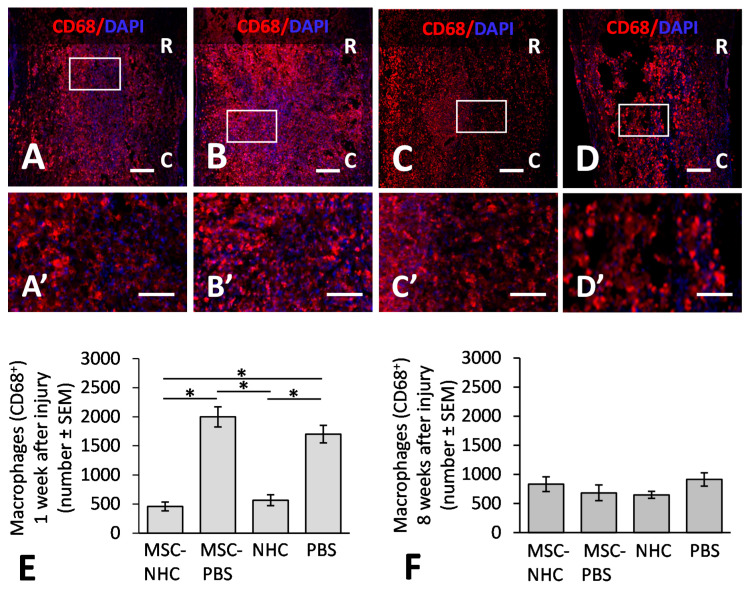
CD68 inflammatory response in the injury site. Photomicrographs showing macrophages stained for CD68 (red), a pan-macrophage marker, and the nucleus marker, DAPI (blue), in the injury site with MSC-NHC (**A**), MSC-PBS (**B**), NHC (**C**), or PBS (**D**) at 1 week after injury. Images in panels (**A’**–**D’**) are enlargements of the outlined area in panels (**A**–**D**), respectively. Scale bar in (**A**,**C**,**D**) is 250 µm, and 200 µm in (**B**). The scale bar in (**A’**–**D’**) is 100 µm. In (**A**–**D**), the rostral (R) and caudal (C) orientation of the horizontal sections are indicated. (**E**) Bar graph showing the average number of CD68+ macrophages in the injury site of each group at 1 week after injury. There are significantly less macrophages in the injury site with MSC-NHC and NHC only compared with MSC-PBS and PBS only. (**F**) Bar graph showing the average number of CD68+ macrophages in the injury site of each group at 8 weeks after injury. The differences in numbers of CD68+ macrophages in the injury site of the four groups were not statistically significant. In both graphs, the asterisks indicate *p* < 0.05, and the bars represent SEM. Abbreviations: DAPI = 4′,6-diamidino-2-phenylindole; MSC = mesenchymal stromal cells; NHC = nanofiber-hydrogel composite; PBS = phosphate-buffered saline; SEM = standard error of the mean.

We assessed the number of macrophages in the injury site at 1 and 8 weeks after injury. Macrophages were present in the injury site at both time points, irrespective of the treatment. Figure 1 shows CD68+ macrophages in the injury site with MSC-NHC (Figure 1A), MSC-PBS (Figure 1B), NHC (Figure 1C), or PBS (Figure 1D) at 1 week after injury. The areas outlined in Figure 1A–D are enlarged in Figure 1A’–D’, respectively. We found 458 ± 75 (mean ± SEM) macrophages in the injury site with MSC-NHC, 1997 ± 174 with MSC-PBS, 565 ± 94 with NHC, and 1699 ± 151 with PBS (Figure 1E). The 4.4-fold decrease in total macrophage count with MSC-NHC compared with MSC-PBS (*p* < 0.05) and the 3.7-fold decrease in macrophages between MSC-NHC and PBS (*p* < 0.05) were significantly different. The 3-fold decrease in macrophages with NHC compared with PBS (*p* < 0.05) and the 3-fold decrease with NHC compared with MSC-PBS (*p* < 0.05) were significantly different. At 8 weeks after injury, there were 833 ± 128 (average ± SEM) macrophages in the injury site with MSC-NHC, 684 ± 135 with MSC-PBS, 649 ± 60 with NHC, and 914 ± 114 with PBS (Figure 1F). The differences in CD68+ macrophage counts at 8 weeks after injury were not significant. The data indicated that early macrophage infiltration in the contusion was attenuated with MSC-NHC or NHC compared with MSC-PBS.

**Figure 2:**
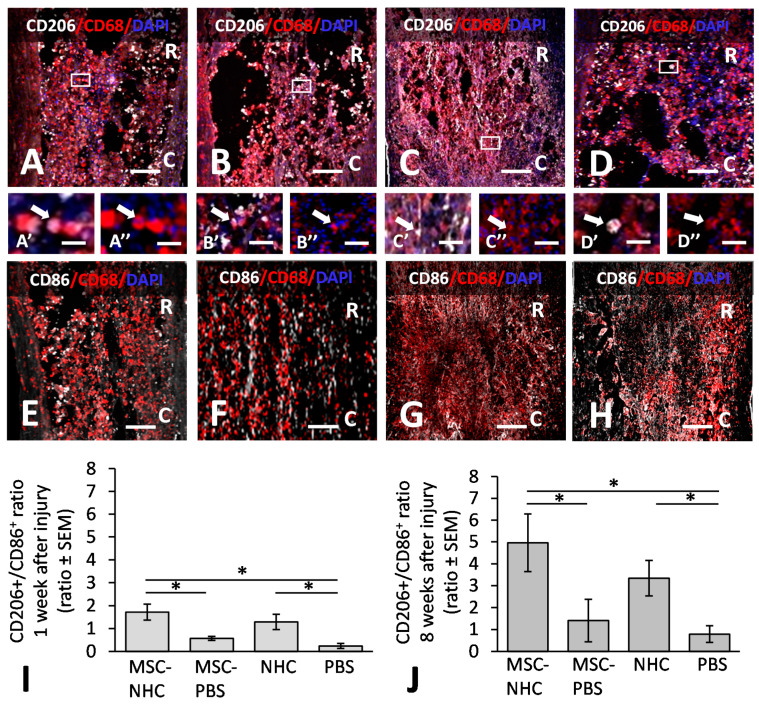
Inflammation polarization ratios in the injury site. Photomicrographs showing macrophages stained for CD206 (white), an anti-inflammatory macrophage marker, CD68 (red), a pan-macrophage marker, and the nucleus marker, DAPI (blue), in the injury site with MSC-NHC (**A**), MSC-PBS (**B**) NHC (**C**), or PBS (**D**) at 8 weeks after injury. Images in panels (**A’**–**D’**) (showing CD206, CD68, and DAPI) and (**A’’**–**D’’**) (showing CD68 and DAPI) are enlargements of the outlined area in panels (**A**–**D**), respectively. Scale bar in (**A**,**C**) is 250 µm, and 200 µm in (**B**,**D**). The scale bar in (**A’**/**A’’**) and (**D’**/**D’’**) is 15 µm, and 25 µm in (**B’**/**B’’**) and (**C’**/**C’’**). In (**A**–**D**), the rostral (R) and caudal (C) orientation of the horizontal sections are indicated. Photomicrographs showing macrophages stained for CD86 (white), a pro-inflammatory macrophage marker, CD68 (red), a pan-macrophage marker, and the nucleus marker, DAPI (blue), in the injury with MSC-NHC (**E**), MSC-PBS (**F**) NHC (**G**), or PBS (**H**) at 8 weeks after injury. The scale bar in (**E**–**H**) is 250 µm. (**I**) Bar graph of the M2/M1 macrophage ratio in the injury site of each group at 1 week after injury. The M2/M1 ratio was significantly higher with MSC-NHC and NHC only than with MSC-PBS and PBS only. There was no statistically significant difference in the M2/M1 ratio of MSC-PBS and NHC only. (**J**) Bar graph of the M2/M1 macrophage ratio in the injury site of each group at 8 weeks after injury. The M2/M1 ratio was significantly higher with MSC-NHC and NHC only than with MSC-PBS and PBS only. There was no statistically significant difference in the M2/M1 ratio of MSC-PBS and NHC only. In both graphs, the asterisks indicate *p* < 0.05, and the bars represent SEM. Abbreviations: DAPI = 4′,6-diamidino-2-phenylindole; MSC = mesenchymal stromal cells; NHC = nanofiber-hydrogel composite; PBS = phosphate-buffered saline; SEM = standard error of the mean.

To assess the relative amount of CD206+ anti-inflammatory, pro-regenerative (M2-like) macrophages vs. CD86+ pro-inflammatory (M1-like) macrophages, we quantified the CD206+/CD86+ ratios at 1 and 8 weeks after injury by analyzing tissue sections stained with antibodies against CD206/CD68 and CD86/CD68. CD206 and CD86 are markers for M2-like and M1-like macrophages, respectively. Figure 2 shows CD206+/CD68+ macrophages in the injury site with MSC-NHC (Figure 2A), MSC-PBS (Figure 2B), NHC (Figure 2C), or PBS (Figure 2D) at 8 weeks after injury. The areas outlined in Figure 2A–D are enlarged in Figure 2A’–D’ and show CD206+/CD68+ macrophages, and Figure 2A’’–D’’ shows the same macrophages stained only for CD68. Moreso, shown are the CD86+/CD68+ macrophages in the injury site with MSC-NHC (Figure 2E), MSC-PBS (Figure 2F), NHC (Figure 2G), or PBS (Figure 2H) at 8 weeks after injury. The areas outlined in Figure 2A–D are enlarged in Figure 2A’–D’ and show CD206+/CD68+ macrophages, and Figure 2A’’–D’’ shows the same macrophages stained only for CD68. At 1 week after injury, we found that the CD206+/CD86+ ratio was 1.72 ± 0.35 in the injury site with MSC-NHC, 0.57 ± 0.09 with MSC-PBS, 1.29 ± 0.33 with NHC, and 0.24 ± 0.11 with PBS (Figure 2I). The CD206+/CD86+ ratio was significantly higher (*p* < 0.05) with MSC-NHC than with MSC-PBS and with PBS. The CD206+/CD86+ ratio was significantly higher with NHC than with PBS (*p* < 0.05). At 8 weeks after injury, the CD206+/CD86+ ratio was 4.96 ± 1.32 in the injury site with MSC-MHC, 1.41 ± 0.97 with MSC-PBS, 3.34 ± 0.81 with NHC, and 0.79 ± 0.38 with PBS (Figure 2J). At this time point, the CD206+/CD86+ ratio was significantly higher (*p* < 0.05) with MSC-NHC than with MSC-PBS or PBS (*p* < 0.05). The results showed that treatment with MSC-NHC compared with MSC-PBS facilitated the polarization of macrophages in the injury site towards the pro-regenerative phenotype.

### 3.2. Injury Site Volume

**Figure 3:**
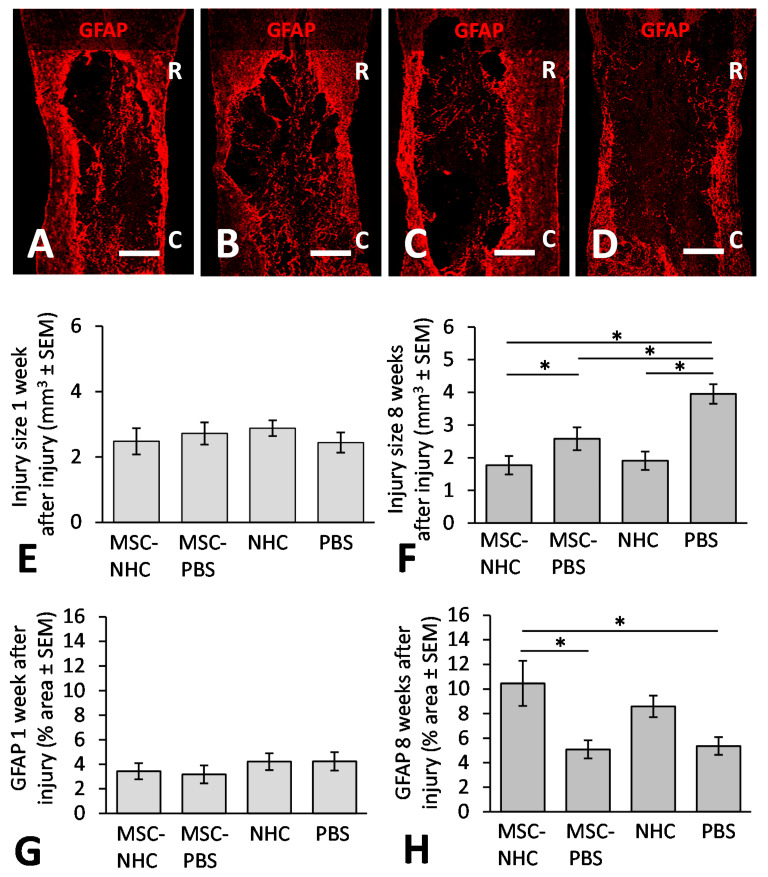
Injury size volume and astrocyte presence. Photomicrographs showing astrocytes stained for GFAP (red) in the injury site with MSC-NHC (**A**), MSC-PBS (**B**), NHC (**C**), or PBS (**D**) at 8 weeks after injury. The injury site was defined by the inner border of the GFAP+ scar. The scale bar in A is 600 µm, and 550 µm in (**B**–**D**). In (**A**–**D**), the rostral (R) and caudal (C) orientation of the horizontal sections are indicated. (**E**) Bar graph showing the volume of the injury site at 1 week after injury. There were no statistically significant differences between groups. (**F**) Bar graph showing the volume of the injury site at 8 weeks after injury. The injury site was significantly smaller with MSC-NHC compared with MSC-PBS or PBS only, with NHC compared with PBS, and with MSC-PBS compared with PBS only. (**G**) Bar graph of the percentage area of the injury site positive for GFAP at 1 week after injury. There were no statistically significant differences in astrocyte presence in the injury site between the groups. (**H**) Bar graph of the percentage area of the injury site positive for GFAP at 8 weeks after injury. There were significantly more astrocytes in the injury site with MSC-NHC compared with MSC-PBS and PBS only. In all graphs, the asterisks indicate *p* < 0.05, and the bars represent SEM. Abbreviations: GFAP = glial fibrillary acidic protein; MSC = mesenchymal stromal cells; NHC = nanofiber-hydrogel composite; PBS = phosphate-buffered saline; SEM = standard error of the mean.

We measured the average volume of the injury site at 1 and 8 weeks after injury. In all animals, an injury site surrounded by a GFAP+ astrocyte scar was discernable at both times after injury. Figure 3 shows the injury site in rats treated with MSC-NHC (Figure 3A), MSC-PBS (Figure 3B), NHC (Figure 3C), or PBS (Figure 3D) at 8 weeks after injury. The volume of the injury site at 1 week after injury was 2.48 ± 0.40 mm^3^ (average ± SEM) with MSC-NHC, 2.72 ± 0.34 mm^3^ with MSC-PBS, 2.88 ± 0.24 mm^3^ with NHC, and 2.44 ± 0.31 mm^3^ with PBS (Figure 3E). There were no statistical differences among these volumes. At 8 weeks after injury, the volume of the injury site was 1.77 ± 0.28 mm^3^ (average ± SEM) with MSC-NHC, 2.58 ± 0.35 mm^3^ with MSC-PBS, 1.91 ± 0.28 mm^3^ with NHC, and 3.95 ± 0.30 mm^3^ with PBS (Figure 3F). Thus, we found a 31 % decrease in injury size with MSC-NHC compared with MSC-PBS (*p* < 0.05), a 55 % decrease in injury size with MSC-NHC compared with PBS (*p* < 0.05), and a 51 % decrease in injury size with NHC compared with PBS (*p* < 0.05). These results showed that MSC-NHC delivery did not result in a significant change in lesion size at 1 week after injury (i.e., 4 days after treatment) over MSC-PBS, NHC, or PBS; however, MSC-NHC and NHC only both limited the secondary injury and yielded a significantly smaller injury compared with MSC-PBS and PBS at week 8.

### 3.3. Astrocytes in the Injury Site

The presence of GFAP+ astrocytes in the injury site was evaluated at 1 and 8 weeks after injury. Figure 3 shows the GFAP-surrounded injury site in rats treated with MSC-NHC (Figure 3A), MSC-PBS (Figure 3B), NHC (Figure 3C), or PBS (Figure 3D) at 8 weeks after injury. At 1 week after injury, GFAP staining occupied 3.43 ± 0.66% (average ± SEM) of the contused segment with MSC-NHC, 3.17 ± 0.73% with MSC-PBS, 4.21 ± 0.68% with NHC, and 4.24 ± 0.75% with PBS (Figure 3G). There were no statistical differences among these measurements. At 8 weeks after injury, GFAP staining occupied 10.46 ± 1.83% (average ± SEM) of the contused segment with MSC-NHC, 5.08 ± 0.4 % with MSC-PBS, 8.59 ± 0.88% with NHC, and 5.36 ± 0.72% with PBS (Figure 3H). The 2.1-fold increase in astrocytes within the injury site with MSC-NHC compared with MSC-PBS (*p* < 0.05) and the 1.9-fold increase in astrocytes within the injury site with MSC-NHC compared with PBS (*p* < 0.05) were statistically significant. The results indicated that treatment with MSC-NHC compared with MSC-PBS resulted in a higher degree of astrocyte presence in the injury site.

### 3.4. Axons in the Injury Site

**Figure 4:**
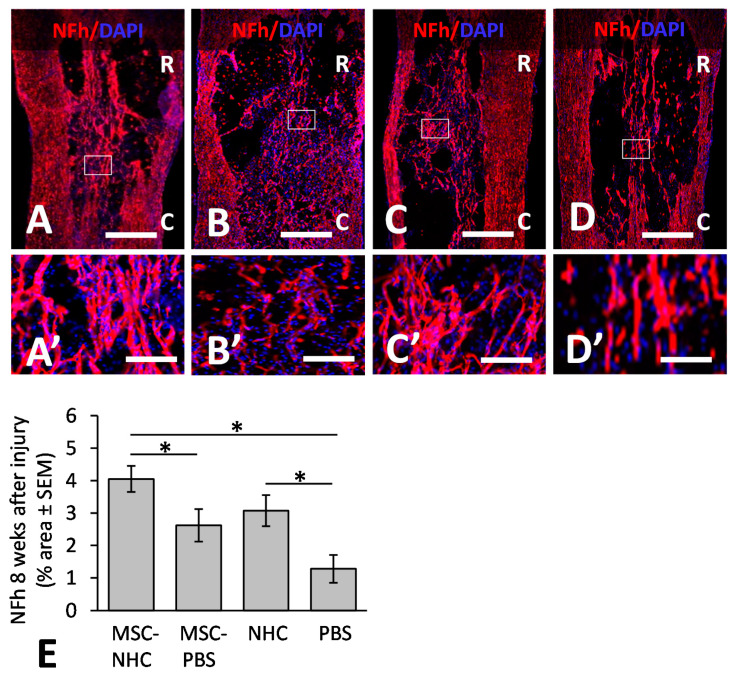
Axon presence in the injury site. Photomicrographs showing axons stained for NFh (red) in the injury site with MSC-NHC (**A**), MSC-PBS (**B**), NHC (**C**), or PBS (**D**) at 8 weeks after injury. Sections were counterstained with the nucleus marker, DAPI (blue). Images in panels (**A’**–**D’**) are enlargements of the outlined area in panels (**A**–**D**), respectively. The scale bar in (**A**–**D** ) is 450 µm. Scale bar in (**A’**,**C’**) is 125 µm, and 115 µm in (**B’**,**D’**). In (**A**–**D**), the rostral (R) and caudal (C) orientation of the horizontal sections are indicated. (**E**) Bar graph of the percentage area of injury site positive for NFh at 8 weeks after injury. There were significantly more NFh+ axons in the injury site with MSC-NHC compared with MSC-PBS and PBS only. Asterisks indicates *p* < 0.05. Bars in the graph represent SEM. Abbreviations: DAPI = 4′,6-diamidino-2-phenylindole; MSC = mesenchymal stromal cells; NFh = neurofilament high molecular weight; NHC = nanofiber-hydrogel composite; PBS = phosphate-buffered saline; SEM = standard error of the mean.

We quantified the presence of axons in the injury site at 8 weeks after injury. Figure 4 shows axons stained for NFh in the injury site treated with MSC-NHC (Figure 4A), MSC-PBS (Figure 4B), NHC (Figure 4C), or PBS (Figure 4D). The areas outlined in Figure 4A–D are enlarged in Figure 4A’–D’, respectively. The NFh+ staining occupied 4.05 ± 0.29% (average ± SEM) of the injury site area with MSC-NHC, 2.62 ± 0.28% with MSC-PBS, 3.07 ± 0.33% with NHC, and 1.28 ± 0.43% with PBS (Figure 4E). The 55% increase in NFh+ axons within the injury site with MSC-NHC compared with MSC-PBS was statistically significant (*p* < 0.05). The 140% increase in NFh+ axons within the injury site with NHC compared with PBS was statistically significant (*p* < 0.05). These results showed that treatment with MSC-NHC compared with MSC-PBS resulted in an increased axon presence in the injury site. Pearson’s correlation analysis showed a strong positive association between the axons and astrocytes (r^2^ = 0.56) in the injury site.

### 3.5. MSC Transplant Survival

**Figure 5:**
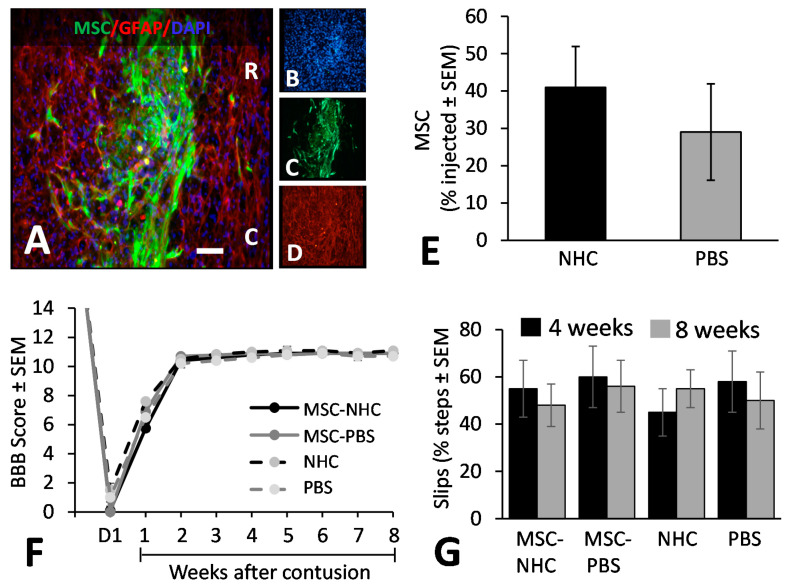
MSC transplant survival and hindlimb function. (**A**) Photomicrograph showing MSC (green), astrocytes (red, anti-GFAP), and cell nuclei (blue, DAPI) in the injury site with MSC-NHC at 1 week after injury. The scale bar in (**A**) is 180 µm. Scale bar in (**B**–**D**) is 550 µm. In (**A**), the rostral (R) and caudal (C) orientation of the horizontal section are indicated. (**E**) Bar graph showing the number of MSCs present in the injury site 1 week after injury. The difference in number between groups was not significant. (**F**) Line graph showing the BBB scores of the experimental groups on day 1 (d1) after injury and weekly thereafter. Differences between groups per time point were not statistically significant. In both graphs, the bars represent SEM. (**G**) Bar graph showing the total slips as a percentage of the total steps made by the rats to cross the horizontal ladder at 4 and 8 weeks after injury. Differences between groups per time point were not statistically significant. In all graphs, the bars represent SEM. Abbreviations: BBB = Basso, Beattie, Bresnahan; d = day; DAPI = 4′,6-diamidino-2-phenylindole; GFAP = glial fibrillary acidic protein; MSC = mesenchymal stromal cells; NHC = nanofiber-hydrogel composite; PBS = phosphate-buffered saline; SEM = standard error of the mean.

We examined transplanted MSC survival in the injury site at 1 and 8 weeks after injury. GFP+ MSC were identified in the injury site at 1 week after injury (Figure 5A–D), but not at 8 weeks after injury. At 1 week after injury, the relative MSC survival, in reference to the number of MSCs injected, was not significantly different between the group with NHC (MSC-NHC; 41 ± 11%; average ± SEM) and the group without NHC (MSC-PBS; 29 ± 13%) (Figure 5E). Our data showed that mixing MSC into NHC compared with PBS prior to injection into damaged nervous tissue in an adult rat contused spinal cord does not significantly affect their survival.

### 3.6. Hindlimb Locomotor Function

We assessed hindlimb motor function using an open-field (BBB) test [29] and hind limb sensorimotor function using a horizontal ladder test [30]. Hindlimb function, as determined by the BBB score, was similar across the groups after injury and after the subsequent treatment. The average BBB score per experimental group at 1 day after injury was 0.1 ± 0.1 (average ± SEM) for rats with MSC-NHC, 0.0 ± 0.0 with MSC-PBS, 1.5 ± 0.5 with NHC, and 1.0 ± 0.5 with PBS (Figure 5F). The average BBB scores gradually increased to reach 10.8 ± 0.1 in rats with MSC-NHC, 10.9 ± 0.1 with MSC-PBS, 11.0 ± 0.1 with NHC, and 10.6 ± 0.2 with PBS (Figure 5F) at 4 weeks after injury. These scores were maintained for the following weeks until the 8-week endpoint. Hindlimb sensorimotor function as assessed on the horizontal ladder was similar among all experimental groups at 4 and 8 weeks after injury (Figure 5G).

## 4. Discussion

The repair potential of MSC-NHC was evaluated in a model of spinal cord contusion. A contusion is the prevalent mechanism of SCI in the clinic. We found that the early inflammatory response in the contused spinal cord of adult rats was substantially attenuated by treatment with MSC-NHC or NHC only, but not with MSC-PBS. Macrophages are necessary in the damaged spinal cord nervous tissue for clearance of cellular/tissue debris and the restoration of homeostasis [33]. During the process, macrophages secrete cytotoxic molecules that may contribute to the propagation of nervous tissue damage in the spinal cord [34,35]. Attenuation of the number of macrophages early after SCI is considered to support nervous tissue repair [36], and this notion was substantiated in previous studies (e.g., [33,34]). The observation that treatment with NHC resulted in a reduction in the macrophage number in the contusion site was consistent with previous findings [22]. We anticipated that treatment with MSC-PBS would not cause a decrease in the number of macrophages in the injury site. While studies have reported anti-inflammatory effects of an MSC transplant [15,16,17,18], this particular MSC-mediated effect has not been associated so far with fewer total macrophages in the injured spinal cord.

The absence of a reduction in the macrophage number in the contusion site with MSC-PBS treatment combined with the decrease in the macrophage number with MSC-NHC and NHC only treatment, points at NHC as the chief mediator of the observed decline in macrophages elicited by MSC-NHC treatment. This proposition would also explain the similar reduction in macrophage numbers by MSC-NHC and NHC only. The ability of NHC to lower the presence of macrophages in an injury site in the spinal cord supports its use as a matrix for cell transplants. Moreover, this finding warrants further investigation of possible mechanisms that underlie the anti-inflammatory effects of NHC.

The inflammatory response in the contused spinal cord of adult rats shifted from a pro-inflammatory-dominant response (low CD206+/CD86+ ratio) towards an anti-inflammatory, pro-regenerative-dominant response (high CD206+/CD86+ ratio) by treatment with MSC-NHC or NHC only, but not with MSC-PBS. A shift in macrophage polarization in the injury site changes their secretome, and thus the molecular environment, in support of tissue remodeling and repair [34,36]. In the injured spinal cord, the chronic pro-inflammatory environment is considered a key contributing factor to continuing nervous tissue damage [33,36]. Our earlier published report on NHC effects on spinal cord repair following SCI supports the finding that NHC modulated macrophages towards a pro-regenerative phenotype [22]. The mechanisms of NHC-mediated macrophage modulation are unknown, but the specific structural design of NHC, including its stiffness and the presence of electrospun fibers, has been proposed to be involved in its immunomodulatory effects [22]. The absent immunomodulatory effect in the contusion site with MSC-PBS treatment was unexpected because previous studies reported that a transplant of MSC exerts such an effect among macrophages present in damaged nervous tissue [7,15,16,17]. It has been suggested that transplanted MSC mediate a change in macrophages towards a pro-regenerative phenotype through paracrine signaling by molecules, such as indoleamine [37] and interleukin-10 [38]. The absence of this effect in our study may be related to factors, such as the severity of the injury or the dose of MSC. In addition, because of the dynamic nature of the inflammatory response, the time of treatment may also play a role in the degree of MSC-mediated modulation of the immune response in the injury site.

The macrophage polarizing effect was greater with MSC-NHC than with MSC-PBS. It is possible that MSC within NHC is less susceptible to injury-derived factors that would decrease their production of molecules directing a macrophage phenotype shift, or alternatively, are more susceptible to factors that would support the secretion of molecules that promote a macrophage phenotype shift. Another possibility is that immunomodulation of the macrophages is promoted by mechanical/physical cues from the NHC besides the soluble factors [39,40]. We can rule out the possibility that MSC survival was improved—which could also result in more molecules that lead to macrophage polarization—because we found similar degrees of survival of MSC in NHC or PBS. The observed interaction between MSC and NHC to trigger the crucial macrophage phenotype shift is a compelling advantage for their combined use for SCI treatment. The critical role of inflammation in SCI [33,41] warrants a future study of mechanisms underlying NHC-mediated modulation of the inflammatory response.

Secondary loss of nervous tissue in the contused spinal cord segment was limited by treatment with MSC-NHC, NHC only, and MSC-PBS. Loss of nervous tissue after the primary damage is a hallmark of SCI. Mechanistically, the primary insult triggers a series of molecular and cellular cytotoxic events that, in concert, cause further nervous tissue damage and degeneration [1,2]. Protecting nervous tissue from secondary damage is an important early line of defense to limit the devastating consequences of SCI [42]. We found that MSC and NHC both have neuroprotective effects that limit injury expansion in the spinal cord. An MSC transplant may exert neuroprotective effects through the secretion of neuroprotective molecules, including neurotrophins (e.g., [12,13,43]. The finding that NHC has neuroprotective effects is in agreement with earlier published observations [22]. The mechanisms of NHC-mediated neuroprotection are under investigation. It is possible that the anti-inflammatory effects of NHC indirectly contribute to its neuroprotective effects [9,34,44].

Neuroprotection in the contused spinal cord by NHC alone and MSC-PBS treatment was similar, while neuroprotection by MSC-NHC treatment was significantly stronger than by MSC-PBS treatment. It is possible that the presence of NHC provided a more favorable environment for the MSC to secrete neuroprotective molecules. There was no significant difference in MSC survival in NHC or PBS, which rules out the possibility that neuroprotective molecules were secreted for a prolonged time due to an extended MSC presence. The significance of neuroprotection in SCI is a compelling argument to use NHC as a transplant matrix for MSC. Future studies will need to focus on elucidating mechanisms underlying the MSC-NHC interactions that support nervous tissue repair.

The presence of axons and astrocytes in the injury site was increased with MSC-NHC. Providing an environment in the injury site that supports the presence of astrocytes and axons is important for the overall repair after spinal cord damage because they may contribute to re-establishing neural tissue and neural connections. Astrocytes and axons are often found closely associated with an injury site in the spinal cord [22]; such a relationship was also observed in the present study. Interestingly, our data suggest that the combined use of MSC and NHC facilitated the establishment of an environment beneficial for astrocytes and axons. Several studies demonstrated that a transplant of MSC supports axon presence in an injury site in the spinal cord [19,20]. The presence of NHC in a spinal cord contusion was shown to result in an increased axon presence [22]. We now show that the MSC and NHC combination resulted in more axons and astrocytes in the injury site compared with either of the single treatments.

Our results showed that an MSC-NHC transplant resulted in an attenuated immune response, macrophage polarization, reduced injury size, and increased axons and astrocytes in the injury site compared with MSC-PBS. These changes did not translate into improved hindlimb function recovery in the used model of contusive SCI, as was shown using the BBB scale and the horizontal ladder walking test. The combinatorial MSC-NHC treatment was investigated for its hypothesized ability to elicit nervous tissue repair in the contused spinal cord. Improved morphological outcomes do not necessarily guarantee improvements in functional outcomes. In this study, the benefit of the observed improvements in morphological outcomes may lie in an increased likelihood and/or efficacy of additional interventions that aim to recover function.

We found that the volume of the injury site was significantly smaller in rats with MSC-PBS compared with PBS, which is in agreement with previous reports [5,6,8,9]. On the other hand, we did not find significant differences in macrophage number, macrophage polarization towards a pro-regenerative phenotype, and astrocyte and axon presence in the injury site between rats with MSC-PBS compared with PBS, which is in discord with prior publications [7,18,19,20]. The absence of these latter repair-supporting effects in rats with MSC-PBS relative to rats with PBS only could be due to the use of MSC derived from bone marrow, which, relative to, for instance, adipose-derived MSC, are characterized by lower in vivo survival and less axon protection in the injured spinal cord [45,46]. Another possibility is that the number of MSC transplanted into the injury may have been too small, compared with previous studies [6,9]. Moreso, the day of transplantation may have affected the outcomes. We injected MSC three days after injury, which is when the inflammatory response reaches its peak [33,34,36].

Our results show that MSC-NHC treatment elicits greater immunomodulation and neuroprotection compared to MSC-PBS or NHC alone. It is possible that the larger effect of MSC-NHC on nervous tissue protection is, at least in part, due to the stronger modulation of the immune response towards a pro-regenerative phenotype. Importantly, our data showed the strongest shift in the immune response and largest neuroprotection with MSC-NHC than with MSC-PBS. These effects were not the result of improved survival of MSC within the NHC matrix. Future studies will need to investigate mechanisms of the relationship between MSC and NHC and explore if other types of repair-mediating cells would also benefit from NHC as their transplant matrix. The observed interactions between MSC and NHC provide an exciting advantage for their combined use for SCI treatment.

## Figures and Tables

**Table 1:** Source, catalog number, and dilution of the used primary antibodies.

Primary Antibody *	Source	Catalog Number	Dilution **
Rabbit anti-GFP	Millipore	AB3080P	1:1000
Mouse anti-GFAP	Sigma	G3893	1:500
Rabbit anti-GFAP	PhosphoSolutions	620-GFAP	1:1000
Mouse anti-ED1 (CD68)	Millipore	MAB1435	1:200
Rabbit anti-NeuN	Millipore	ABN78	1:500
Rabbit anti-CD206	Abcam	Ab64693	1:500
Rabbit anti-CD86	Abcam	Ab53004	1:500
Rabbit anti-NFh	Millipore	AB1991	1:500

* All antibodies are commercially available. Their source and catalog number are listed. ** Dilutions in PBS with 5% normal goat serum and 0.3% Triton X-100.

## Data Availability

The data presented in this study are available on request from the corresponding author.

## References

[1] Hachem L.D., Fehlings M.G. (2021). Pathophysiology of Spinal Cord Injury. Neurosurg. Clin. N. Am..

[2] Hagg T., Oudega M. (2006). Degenerative and spontaneous regenerative processes after spinal cord injury. J. Neurotrauma.

[3] van Niekerk E.A., Tuszynski M.H., Lu P., Dulin J.N. (2016). Molecular and cellular mechanisms of axonal regeneration after spinal cord injury. Mol. Cell. Proteom..

[4] Walsh C.M., Wychowaniec J.K., Brougham D.F., Dooley D. (2021). Functional hydrogels as therapeutic tools for spinal cord injury: New perspectives on immunopharmacological interventions. Pharmacol. Ther..

[5] Cizkova D., Rosocha J., Vanicky I., Jergova S., Cizek M. (2006). Transplants of human mesenchymal stem cells improve functional recovery after spinal cord injury in the rat. Cell. Mol. Neurobiol..

[6] Nandoe Tewarie R.D., Hurtado A., Ritfeld G.J., Rahiem S.T., Wendell D.F., Barroso M.M., Grotenhuis J.A., Oudega M. (2009). Bone marrow stromal cells elicit tissue sparing after acute but not delayed transplantation into the contused adult rat thoracic spinal cord. J. Neurotrauma.

[7] Nakajima H., Uchida K., Guerrero A.R., Watanabe S., Sugita D., Takeura N., Yoshida A., Long G., Wright K.T., Johnson W.E. (2012). Transplantation of mesenchymal stem cells promotes an alternative pathway of macrophage activation and functional recovery after spinal cord injury. J. Neurotrauma.

[8] Quertainmont R., Cantinieaux D., Botman O., Sid S., Schoenen J., Franzen R. (2012). Mesenchymal stem cell graft improves recovery after spinal cord injury in adult rats through neurotrophic and pro-angiogenic actions. PLoS ONE.

[9] Ritfeld G.J., Tewarie R.N., Vajn K., Rahiem S.T., Hurtado A., Wendell D.F., Roos R.A., Oudega M. (2012). Bone marrow stromal cell-mediated tissue sparing enhances functional repair after spinal cord contusion in adult rats. Cell Transpl..

[10] Ritfeld G.J., Rauck B.M., Novosat T.L., Park D., Patel P., Roos R.A., Wang Y.D., Oudega M. (2014). The effect of a polyurethane-based reverse thermal gel on bone marrow stromal cell transplant survival and spinal cord repair. Biomaterials.

[11] Morita T., Sasaki M., Kataoka-Sasaki Y., Nakazaki M., Nagahama H., Oka S., Oshigiri T., Takebayashi T., Yamashita T., Kocsis J.D. (2016). Intravenous infusion of mesenchymal stem cells promotes functional recovery in a model of chronic spinal cord injury. Neurosci.

[12] Liu Y., Dulchavsky D.S., Gao X., Kwon D., Chopp M., Dulchavsky S., Gautam S.C. (2006). Wound repair by bone marrow stromal cells through growth factor production. J. Surg. Res..

[13] Nakano N., Nakai Y., Seo T.B., Yamada Y., Ohno T., Yamanaka A., Nagai Y., Fukushima M., Suzuki Y., Nakatani T. (2010). Characterization of conditioned medium of cultured bone marrow stromal cells. Neurosci. Lett..

[14] Ritfeld G.J., Patel A., Chou A., Novosat T.L., Castillo D.G., Roos R.A., Oudega M. (2015). The role of brain-derived neurotrophic factor in bone marrow stromal cell-mediated spinal cord repair. Cell Transpl..

[15] Tomchuck S.L., Zwezdaryk K.J., Coffelt S.B., Waterman R.S., Danka E.S., Scandurro A.B. (2008). Toll-like receptors on human mesenchymal stem cells drive their migration and immunomodulating responses. Stem. Cells.

[16] Shin T., Ahn M., Moon C., Kim S., Sim K.B. (2013). Alternatively activated macrophages in spinal cord injury and remission: Another mechanism for repair?. Mol. Neurobiol..

[17] He X., Wang H., Jin T., Xu Y., Mei L., Yang J. (2016). TLR4 activation promotes bone marrow MSC proliferation and osteogenic differentiation via wnt3a and wnt5a signaling. PLoS ONE.

[18] Cofano F., Boido M., Monticelli M., Zenga F., Ducati A., Vercelli A., Garbossa D. (2019). Mesenchymal stem cells for spinal cord injury: Current options, limitations, and future of cell therapy. Int. J. Mol. Sci..

[19] Okuda A., Horii-Hayashi N., Sasagawa T., Shimizu T., Shigematsu H., Iwata E., Morimoto Y., Masuda K., Koizumi M., Akahane M. (2017). Bone marrow stromal cell sheets may promote axonal regeneration and functional recovery with suppression of glial scar formation after spinal cord transection injury in rats. J. Neurosurg. Spine.

[20] Lin L., Lin H., Bai S., Zheng L., Zhang X. (2018). Bone marrow mesenchymal stem cells (BMSCs) improved functional recovery of spinal cord injury partly by promoting axonal regeneration. Neurochem. Int..

[21] Nandoe Tewarie R.D., Hurtado A., Levi A.D., Grotenhuis J.A., Oudega M. (2006). Bone marrow stromal cells for repair of the spinal cord: Towards clinical application. Cell Transplant.

[22] Li X., Zhang C., Haggerty A.E., Yan J., Lan M., Seu M., Yang M., Marlow M.M., Maldonado-Lasunción I., Cho B. (2020). The effect of a nanofiber-hydrogel composite on neural tissue repair and regeneration in the contused spinal cord. Biomaterials.

[23] Ozawa H., Matsumoto T., Ohashi T., Sato M., Kokubun S. (2001). Comparison of spinal cord gray matter and white matter softness: Measurement by pipette aspiration method. J. Neurosurg..

[24] Ware T., Simon D., Arreaga-Salas D.E., Reeder J., Rennaker R., Keefer E.W., Voit W. (2012). Fabrication of responsive, softening neural interfaces. Adv. Funct. Mater..

[25] Li X., Cho B., Martin R., Seu M., Zhang C., Zhou Z., Choi J.S., Jiang X., Chen L., Walia G. (2019). Nanofiber-hydrogel composite mediated angiogenesis for soft tissue reconstruction. Sci. Transl. Med..

[26] Azizi S.A., Stokes D., Augelli B.J., DiGirolamo C., Prockop D.J. (1998). Engraftment and migration of human bone marrow stromal cells implanted in the brains of albino rats—Similarities to astrocyte grafts. Proc. Natl. Acad. Sci. USA.

[27] Scheff S.W., Rabchevsky A.G., Fugaccia I., Main J.A., Lumpp J.E. (2003). Experimental modeling of spinal cord injury: Characterization of a force-defined injury device. J. Neurotrauma.

[28] Rauck B.M., Novosat T.L., Oudega M., Wang Y. (2015). Biocompatibility of a coacervate-based controlled release system for protein delivery to the injured spinal cord. Acta Biomater..

[29] Basso M., Beattie M.S., Bresnahan J.C. (1995). A sensitive and reliable locomotor rating scale for open field testing in rats. J. Neurotrauma.

[30] Kunkel-Bagden E., Dai H.N., Bregman B.S. (1993). Methods to assess the development and recovery of locomotor function after spinal cord injury in rats. Exp. Neurol..

[31] Metz G.A., Whishaw I.Q. (2002). Drug-induced rotation intensity in unilateral dopamine-depleted rats is not correlated with end point or qualitative measures of forelimb or hindlimb motor performance. Neuroscience.

[32] Noller C.M., Boulina M., McNamara G., Szeto A., McCabe P.M., Mendez A.J. (2016). A practical approach to quantitative processing and analysis of small biological structures by fluorescent imaging. J. Biomol. Tech..

[33] Gensel J.C., Zhang B. (2015). Macrophage activation and its role in repair and pathology after spinal cord injury. Brain Res..

[34] Kigerl K.A., Gensel J.C., Ankeny D.P., Alexander J.K., Donnelly D.J., Popovich P.G. (2009). Identification of two distinct macrophage subsets with divergent effects causing either neurotoxicity or regeneration in the injured mouse spinal cord. J. Neurosci..

[35] Kroner A., Greenhalgh A.D., Zarruk J.G., Passos Dos Santos R., Gaestel M., David S. (2014). TNF and increased intracellular iron alter macrophage polarization to a detrimental M1 phenotype in the injured spinal cord. Neuron.

[36] Maldonado-Lasunción I., Verhaagen J., Oudega M. (2018). Mesenchymal stem cell-macrophage choreography supporting spinal cord repair. Neurotherapeutics.

[37] Ge W., Jiang J., Arp J., Liu W., Garcia B., Wang H. (2010). Regulatory T-cell generation and kidney allograft tolerance induced by mesenchymal stem cells associated with indoleamine 2,3-dioxygenase expression. Transplantation.

[38] Wheeler K.C., Jena M.K., Pradhan B.S., Nayak N., Das S., Hsu C.D., Wheeler D.S., Chen K., Nayak N.R. (2018). VEGF may contribute to macrophage recruitment and M2 polarization in the decidua. PLoS ONE.

[39] Garg K., Pullen N.A., Oskeritzian C.A., Ryan J.J., Bowlin G.L. (2013). Macrophage functional polarization (M1/M2) in response to varying fiber and pore dimensions of electrospun scaffolds. Biomaterials.

[40] McWhorter F.Y., Wang T., Nguyen P., Chung T., Liu W.F. (2013). Modulation of macrophage phenotype by cell shape. Proc. Natl. Acad. Sci. USA.

[41] Popovich P.G., Guan Z., McGaughy V., Fisher L., Hickey W.F., Basso D.M. (2002). The neuropathological and behavioral consequences of intraspinal microglial/macrophage activation. J. Neuropathol. Exp. Neurol..

[42] Fiani B., Kondilis A., Soula M., Tao A., Alvi M.A. (2021). Novel methods of necroptosis inhibition for spinal cord injury using translational research to limit secondary injury and enhance endogenous repair and regeneration. Neurospine.

[43] Labrador-Velandia S., Alonso-Alonso M.L., Di Lauro S., García-Gutierrez M.T., Srivastava G.K., Pastor J.C., Fernandez-Bueno I. (2019). Mesenchymal stem cells provide paracrine neuroprotective resources that delay degeneration of co-cultured organotypic neuroretinal cultures. Exp. Eye Res..

[44] Krupa P., Vackova I., Ruzicka J., Zaviskova K., Dubisova J., Koci Z., Turnovcova K., Urdzikova L.M., Kubinova S., Rehak S. (2018). The effect of human mesenchymal stem cells derived from Wharton’s Jelly in spinal cord injury: Treatment is dose-dependent and can be facilitated by repeated application. Int. J. Mol. Sci..

[45] Zhou Z., Chen Y., Zhang H., Min S., Yu B., He B., Jin A. (2013). Comparison of mesenchymal stromal cells from human bone marrow and adipose tissue for the treatment of spinal cord injury. Cytotherapy.

[46] Takahashi A., Nakajima H., Uchida K., Takeura N., Honjoh K., Watanabe S., Kitade M., Kokubo Y., Johnson W.E.B., Matsumine A. (2018). Comparison of Mesenchymal Stromal Cells Isolated from Murine Adipose Tissue and Bone Marrow in the Treatment of Spinal Cord Injury. Cell Transpl..

